# High-Resolution Snapshot Multispectral Imaging System for Hazardous Gas Classification and Dispersion Quantification

**DOI:** 10.3390/mi17010112

**Published:** 2026-01-14

**Authors:** Zhi Li, Hanyuan Zhang, Qiang Li, Yuxin Song, Mengyuan Chen, Shijie Liu, Dongjing Li, Chunlai Li, Jianyu Wang, Renbiao Xie

**Affiliations:** 1 Hangzhou Institute for Advanced Study, University of Chinese Academy of Sciences, Hangzhou 310024, China; lizhi23@mails.ucas.ac.cn (Z.L.);; 2 University of Chinese Academy of Sciences, Beijing 100049, China; 3 Shanghai Institute of Technical Physics, Chinese Academy of Sciences, Shanghai 200083, China; 4 Shanghai Micro Electronics Equipment (Group) Co., Ltd., Shanghai 201203, China

**Keywords:** industrial monitoring, gas leak detection, snapshot multispectral imaging system (SMIS), high spatial resolution, microlens array (MLA), long-wave infrared (LWIR)

## Abstract

Real-time monitoring of hazardous gas emissions in open environments remains a critical challenge. Conventional spectrometers and filter wheel systems acquire spectral and spatial information sequentially, which limits their ability to capture multiple gas species and dynamic dispersion patterns rapidly. A High-Resolution Snapshot Multispectral Imaging System (HRSMIS) is proposed to integrate high spatial fidelity with multispectral capability for near real-time plume visualization, gas species identification, and concentration retrieval. Operating across the 7–14 μm spectral range, the system employs a dual-path optical configuration in which a high-resolution imaging path and a multispectral snapshot path share a common telescope, allowing for the simultaneous acquisition of fine two-dimensional spatial morphology and comprehensive spectral fingerprint information. Within the multispectral path, two 5×5 microlens arrays (MLAs) combined with a corresponding narrowband filter array generate 25 distinct spectral channels, allowing concurrent detection of up to 25 gas species in a single snapshot. The high-resolution imaging path provides detailed spatial information, facilitating spatio-spectral super-resolution fusion for multispectral data without complex image registration. The HRSMIS demonstrates modulation transfer function (MTF) values of at least 0.40 in the high-resolution channel and 0.29 in the multispectral channel. Monte Carlo tolerance analysis confirms imaging stability, enabling the real-time visualization of gas plumes and the accurate quantification of dispersion dynamics and temporal concentration variations.

## 1. Introduction

Due to the progression of industrialization, major hazard facilities often operate continuously under harsh conditions, such as high temperatures, elevated pressures, and intense flow rates [1,2]. Such environments substantially increase the likelihood of leaks involving flammable, toxic, or greenhouse gases [3,4]. These leaks can lead to fires, explosions, and toxic-exposure incidents, frequently causing casualties and significant property damage. In inaccessible or hazardous areas, rapid non-contact monitoring of multiple gas species is crucial [5,6]. Remote infrared spectroscopic imaging provides a practical approach to this requirement by enabling gas identification through characteristic absorption features. The longwave infrared (LWIR) region, spanning approximately 7–14 μm, is particularly important because many hazardous gases exhibit distinct absorption bands within this range [7,8,9]. Passive infrared imaging exploits the selective absorption of background radiation by gas plumes to achieve visualization. When combined with multispectral imaging [10,11], multiband radiance data enable the identification of multiple gas species [12,13].

Fourier transform and grating spectrometers, filter wheel assemblies, and liquid crystal tunable filters have been widely employed for gas detection and analysis [14,15]. The FIRST hyperspectral imager based on Fourier transform infrared spectroscopy (FTIR) [16,17,18,19,20,21,22] provides high spectral resolution but requires extensive computation and cannot capture rapidly evolving gas plumes in real time. With a focal length of 86 mm and an F-number of 2, the optical configuration achieves a spatial resolution of 0.35 mrad. However, the limited signal-to-noise ratio (SNR) constrains the detection of weakly absorbing gases [23,24,25]. The Second Sight multispectral imaging system, utilizing a filter wheel mechanism, attains a spatial resolution of 0.71 mrad with a 15 μm pixel pitch and a 7 mm focal length. An F-number of 4 restricts optical throughput, thereby reducing SNR and overall sensitivity. Additionally, the limitation to six spectral channels and the image artifacts caused by high-speed filter switching restrict the rapid and simultaneous observation of multiple gas species [26,27,28]. Olbrycht and Kałuża demonstrated a gas imaging approach based on a 640 × 480 uncooled ${\mathrm{VO}}_{x}$ microbolometer thermographic camera with a 35 mm, f/1 lens. The spatial resolution of the camera is approximately 0.24 mrad. A single interference bandpass filter, mounted in front of the lens, attenuates the in-band irradiance, resulting in increased noise levels. Gas species can only be detected sequentially through mechanical filter replacement, precluding simultaneous multi-gas observation. It should also be noted that absorption overlap between the target gas and atmospheric constituents may introduce cross-sensitivity, which becomes more significant for longer propagation distances [29]. Providence Photonics developed a snapshot multispectral imaging method in which four adjacent pixels (arranged in a 2 × 2 pattern) are assigned to different spectral bands, enabling concurrent detection of up to four gas species [30]. The temporal separation between spectral and spatial data acquisition limits the fast and accurate identification of numerous gases exhibiting distinct absorption features and obstructs real-time tracking of their diffusion dynamics and spatial evolution. Low spatial resolution and restricted sensitivity remain persistent constraints in existing instrumentation, indicating the necessity for an imaging methodology capable of achieving high-resolution, high-sensitivity, and real-time observation of gas distribution and concentration variations.

A High-Resolution Snapshot Multispectral Imaging System (HRSMIS) is proposed in this paper to address the aforementioned limitations. The instrument combines high spatial resolution with multispectral imaging capability, enabling near-real-time visualization, identification, and quantitative retrieval of multiple gas species. The HRSMIS employs a dual-path, parallel optical configuration comprising a multispectral imaging path and a high-resolution imaging path, both of which are fed by a common front telescope with an F-number of 1. In the multispectral path, two 5×5 microlens arrays (MLAs) coupled with a narrowband filter array provide 25 discrete spectral channels, each corresponding to a specific sub-aperture and wavelength band. HRSMIS employs a MLA, but its operating principle differs fundamentally from that of light-field cameras. In light-field imaging, the MLA samples the light field by associating each microlens with a spatial sampling location, such that the resulting micro-image maps different portions of the main lens aperture onto distinct pixels. As a result, pixel positions within each micro-image correspond to different sub-apertures, enabling angular sampling that supports digital refocusing and three-dimensional reconstruction [31]. In contrast, the MLA in HRSMIS is co-registered with a narrowband filter array, with each microlens paired to an individual filter element. Such a configuration partitions the same field of view (FOV) into multiple spectral imaging channels within a single exposure. Each channel forms a two-dimensional image of the same scene, while the spectral response is defined by the corresponding filter element, leading to distinct center wavelengths across channels. Accordingly, snapshot imaging in HRSMIS emphasizes spectral sampling and simultaneous multi-band acquisition rather than the angular sampling characteristic of light-field imaging. Within a single capture, the system records the absorption characteristics of up to 25 hazardous gases across the 7–14 μm spectral range, while the high-resolution path performs broadband imaging within the same range, achieving a spatial resolution of 0.13 mrad. The system can be combined with super-resolution fusion techniques to generate high-spatial-resolution multispectral imagery of the detected gases. Because both optical paths are aligned within a single telescope aperture, they maintain identical viewing geometries, which eliminates parallax between images and eliminates the need for complex registration procedures. The HRSMIS enables the concurrent identification of multiple gas species, detailed mapping of their spatial distributions, and sensitive measurement of scene radiance within a single millisecond-scale acquisition. Continuous operation supports video-rate monitoring of temporal concentration variations and diffusion dynamics of gaseous emissions.

The structure of this paper is as follows. Section 2 introduces the overall architecture and gas-detection principles of the HRSMIS. Section 3 presents the optical design, including the design methodology, key parameters, and detailed configurations of the telescope, the high-resolution imaging subsystem, and the multispectral imaging subsystem. Section 4 evaluates the imaging performance at both subsystem and integrated-system levels. Section 5 presents the results of the tolerance analysis. Section 6 discusses the analytical results, and Section 7 concludes the paper.

## 2. HRSMIS Principle

### 2.1. Architectural Overview

**Figure 1:**
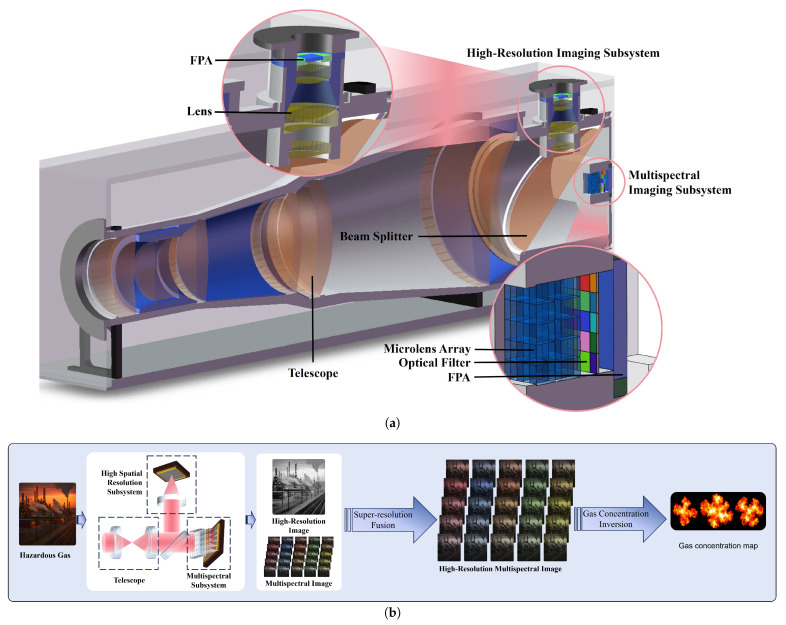
The HRSMIS system. (**a**) Schematic diagram of HRSMIS system composition. (**b**) Schematic of HRSMIS operating principle.

Figure 1a illustrates the overall structure of the HRSMIS, which integrates an optical assembly, two uncooled detectors, and an image-processing module. The instrument directly observes the target field, and its operational principle for hazardous-gas monitoring is shown in Figure 1b. At the core of the HRSMIS is an optical assembly composed of two coordinated imaging subsystems, namely a high-resolution imaging subsystem and a multispectral imaging subsystem, both receiving incident radiation through the same fore-optics to achieve co-axial observation without parallax and to enhance the SNR. The fore-optics operates in a collimated configuration, allowing parallel beams to enter the downstream subsystems. A beam splitter then divides the collimated flux and directs it along separate optical paths.

HRSMIS achieves spectral separation through a narrowband filter array, in which each multispectral sub-aperture is coupled to a dedicated filter centered at a distinct wavelength. Each channel provides a spectrally isolated narrowband measurement at the FPA, with negligible spectral overlap, owing to the 80 nm filter bandwidth and an out-of-band optical density of OD4. As a result, the FPA does not record spectrally multiplexed signals, and gas mixtures do not cause spectral mixing at the detector. Such spectral channelization enables the simultaneous acquisition of single-band images of an identical scene across all channels, thereby facilitating subsequent gas analysis. The high-spatial-resolution subsystem and the multispectral subsystem acquire data simultaneously over the same scene. Radiometric calibration is first performed using the variable-temperature blackbody, after which super-resolution fusion integrates the high-resolution spatial information with the 5 × 5 single-band measurements to produce a high-resolution multispectral result. The reconstruction yields 25 high-spatial-resolution, narrowband images that cover the same imaged area. Each snapshot takes only a few tens of milliseconds to finish. Gas concentration is retrieved from the differential radiance between two successive frames at the focal plane array (FPA).

After data acquisition by the two HRSMIS subsystems, a neural-network-guided super-resolution reconstruction is applied to generate high-spatial-resolution multispectral imagery. In the end-to-end framework, the network takes the high-resolution image from the high-resolution channel, along with the sub-aperture images from the multispectral subsystem, as inputs and outputs high-resolution multispectral sub-aperture images. The overall framework consists of two components: a super-resolution reconstruction branch based on a pretrained ESRGAN model and a high-resolution guidance branch. The high-resolution image serves as a spatial prior, with its fine-scale structural details constraining and guiding the per-channel single-image super-resolution of each multispectral sub-aperture image. As a result, approximately fivefold super-resolved multispectral sub-aperture images are obtained, enabling the reconstruction of a high-spatial-resolution multispectral output.

### 2.2. Image Formation Model

**Figure 2:**
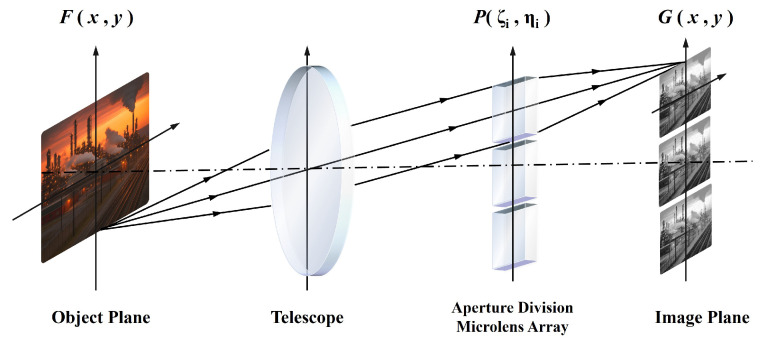
The relationship between image and object in the aperture division MLA imaging system.

Considering the 5×5 spatial configuration of the MLA in the multispectral imaging subsystem of the HRSMIS, which performs sub-aperture imaging of the same target scene rather than single-aperture imaging, it is essential to establish an imaging model and analyze the resulting imaging characteristics. Although the lens groups are primarily designed and optimized using geometrical ray tracing, the subimage size, separation, and interchannel crosstalk in the MLA-based multispectral path are ultimately constrained by diffraction at the MLA–sensor plane. Therefore, the following Fourier-optics model is introduced to provide analytical constraints for selecting the MLA pitch, focal length, and F-number, which are then implemented and verified in the subsequent geometrical optical design. Figure 2 illustrates the correlation between the intensity distribution at the object plane and that at the image plane for an aperture-division MLA. In Fourier optics, the imaging process can be approximated as a linear shift-invariant (LSI) system under incoherent illumination within a local isoplanatic region. Accordingly, the detector-plane intensity is given by the convolution of the object-plane intensity with the system point spread function (PSF) PSF(x,y) [32,33,34]:

(1) G(x,y)=F(x,y)∗PSF(x,y).

Here, PSF(x,y) denotes the system’s intensity response on the image plane to a point source located in the object plane. In incoherent diffraction-limited imaging, the PSF is given by the squared magnitude of the coherent impulse response, which is proportional to the Fourier transform of the pupil function. The pupil function of the central channel is defined as outlined in Equation (2).

In incoherent limited by diffraction imaging, the PSF is defined as the squared magnitude of the Fourier transform of the pupil function. Define the pupil function of the central channel as outlined in Equation (2).

(2) $$P_{0}\left(\xi ,\eta \right)=\mathrm{rect}\left(\frac{\xi }{l_{x}}\right)\mathrm{rect}\left(\frac{\eta }{l_{y}}\right),$$

Here, ξ and η represent the coordinates in the pupil plane, and $l_{x}$ and $l_{y}$ indicate the dimensions of the subaperture within the pupil plane.

The associated separable one-dimensional Fourier transforms are articulated in Equation (3).

(3) $$\left\{\begin{array}{c} F\left\{\mathrm{rect}\left(\frac{\xi }{l_{x}}\right)\right\}=l_{x}\mathrm{sinc}\left(l_{x}f_{x}\right),\\ F\left\{\mathrm{rect}\left(\frac{\eta }{l_{y}}\right)\right\}=l_{y}\mathrm{sinc}\left(l_{y}f_{y}\right), \end{array}\right.$$

In this context, $\mathrm{sinc}\left(u\right)=\frac{\sin(\pi u)}{\pi u}$, and the normalized frequency-space coordinates are specified as $f_{x}=x/\left(\lambda f\right)$ and $f_{y}=y/\left(\lambda f\right)$, where λ represents the wavelength and *f* denotes the focal length.

The central channel’s intensity PSF is

(4) $${\mathrm{PSF}}_{0}(x,y)\propto |\mathrm{sinc}\left(l_{x}f_{x}\right){|}^{2}|\mathrm{sinc}\left(l_{y}f_{y}\right){|}^{2}.$$

Equation (4) indicates that the PSF extent scales with wavelength and is inversely proportional to the subaperture dimensions. This scaling provides a quantitative basis for setting the minimum separation between adjacent subimages on the detector to suppress overlap and interchannel crosstalk.

For a decentered subaperture labeled *i*, the pupil function $P_{i}\left(\xi ,\eta \right)$ is obtained by translating Equation (2):

(5) $$P_{i}\left(\xi ,\eta \right)=\mathrm{rect}\left(\frac{\xi -\xi_{i}}{l_{x}}\right)\mathrm{rect}\left(\frac{\eta -\eta_{i}}{l_{y}}\right),$$

where $\left(\xi_{i},\eta_{i}\right)$ is the pupil-plane offset relative to the central channel. Its image-plane complex amplitude is

(6) $$h_{i}\left(x,y\right)\;\propto \;\mathrm{sinc}\left(l_{x}f_{x}\right)\mathrm{sinc}\left(l_{y}f_{y}\right)\;\exp\left[-j2\pi \left(\xi_{i}f_{x}+\eta_{i}f_{y}\right)\right],$$

So, decentering introduces only a linear phase factor. Therefore, the intensity PSF of each channel is

(7) $${\mathrm{PSF}}_{i}\left(x,y\right)=|h_{i}\left(x,y\right){|}^{2}=|\mathrm{sinc}\left(l_{x}f_{x}\right)\mathrm{sinc}\left(l_{y}f_{y}\right){|}^{2}={\mathrm{PSF}}_{0}\left(x,y\right),$$

This equality holds at a given wavelength when subaperture decentering introduces only a linear phase term and the differences in aberrations and vignetting among subapertures are negligible; practical deviations are evaluated in the subsequent ray-tracing-based image quality analysis. The corresponding optical transfer function is likewise independent of $\left(\xi_{i},\eta_{i}\right)$, and can therefore be expressed as ${\mathrm{OTF}}_{i}={\mathrm{OTF}}_{0}$.

The affine mapping for the image centroid of channel *i* on the image plane is

(8) $$\left(\begin{array}{c} u_{i}\\ v_{i} \end{array}\right)=M\left(\begin{array}{c} \xi_{i}\\ \eta_{i} \end{array}\right)+\left(\begin{array}{c} u_{0}\\ v_{0} \end{array}\right),$$

where $M\in R^{2\times 2}$ and $\left(u_{0},v_{0}\right)$ are obtained through system calibration.

The theoretical relationships established above delineate the feasible ranges for the MLA pitch, the focal length, and the F-number necessary to preserve uniform PSFs across channels, mitigate interchannel crosstalk, and guarantee consistent subimage placement. The following section presents the parameter choices guided by these constraints, and the resulting performance is then implemented and verified through geometrical optical design and optimization in the subsequent sections.

## 3. HRSMIS Optical System Design

### 3.1. Parameter Constraints

In the design of the MLA of HRSMIS, to prevent crosstalk between neighboring subchannels, the usable pixel counts for each subchannel must comply with

(9) $$n_{y}\leq \left\lfloor \frac{N_{y}}{M}-2m\right\rfloor ,\;n_{x}\leq \left\lfloor \frac{N_{x}}{M}-2m\right\rfloor ,$$

Here, $N_{x}$ and $N_{y}$ denote the maximum usable pixel counts along the *x* and *y* axes for a single subchannel on the image plane; $L_{x}$ and $L_{y}$ represent the dimensions of the detector along the *x* and *y* axes; *M* indicates the number of subchannels per axis; and *G* refers to the guard band between adjacent subimages, generally ranging from 3 to 5 pixels to reduce interchannel crosstalk. The existing configuration permits a maximum operational grid of $N_{x}\times N_{y}$ pixels per subchannel.

The camera focal length *f* is ascertained from the spatial resolution requirement through geometric similarity.

(10) $$\frac{\mathrm{GSD}}{R}=\frac{d}{f_{c}},$$

where GSD denotes the ground sampling distance, *R* represents the working distance, *d* signifies the detector pixel size, and $f_{c}$ indicates the camera focal length. For FPAs with a pixel size of 15μm and a detection distance of 1 km, the high-resolution channel employs a focal length of 110 mm, while each multispectral subchannel utilizes a focal length of 16.5 mm.

Let $L_{\mathrm{off}}$ represent the atmospheric radiance at the entrance pupil of the HRSMIS optical system from the background when the line of sight does not intersect the hazardous gases, and let $L_{\mathrm{on}}$ denote the entrance pupil radiance when the line of sight intersects the gas plume and traverses an effective absorption path length $l_{\mathrm{eff}}$ within the gas cloud; in the absence of the gas plume, both values are equivalent. In practical scenarios, the separation between the gas plume and the background along the line of sight is minimal, so attenuation and emission over that brief segment can be neglected. The imaging distance *R* is used only for geometrical imaging and spatial resolution budgeting, and it is treated independently from the absorption modeling. The gas transmittance $\tau_{\mathrm{gas}}$ is determined by the absorption that occurs within the gas plume, which is parameterized by $l_{\mathrm{eff}}$ or, equivalently, by the line of sight integrated concentration. The associated radiative transfer equations are

(11) $$\begin{array}{cc} L_{\mathrm{on}}\left(\lambda_{i}\right) & =L_{\mathrm{bg}}\left(\lambda_{i}\right)\tau_{\mathrm{gas}}\left(\lambda_{i}\right)+L_{\mathrm{gas}}\left(\lambda_{i}\right),\\ L_{\mathrm{off}}\left(\lambda_{i}\right) & =L_{\mathrm{bg}}\left(\lambda_{i}\right),\\ L_{\mathrm{gas}}\left(\lambda_{i}\right) & =B\left(\lambda_{i},T_{\mathrm{gas}}\right)\left[1-\tau_{\mathrm{gas}}\left(\lambda_{i}\right)\right], \end{array}$$

where $L_{\mathrm{bg}}$ is the background radiance at the entrance pupil, $L_{\mathrm{gas}}$ is the gas self-emission at the entrance pupil, $B(\lambda ,T_{\mathrm{gas}})$ is the Planck radiance of the gas, and $\tau_{\mathrm{gas}}$ is the gas transmittance determined by absorption within the gas cloud.

The radiance difference mapped to the detector is

(12) $$\Delta L=\tau_{\mathrm{opt}}\left(L_{\mathrm{off}}-L_{\mathrm{on}}\right)=\tau_{\mathrm{opt}}\left(1-\tau_{\mathrm{gas}}\right)\left(L_{\mathrm{bg}}-B\right),$$

where the overall optical transmittance is given by $\tau_{\mathrm{opt}}=\tau_{f}\tau_{o}$.

The spectral power reaching one pixel in a single spectral channel is

(13) $$P_{\lambda ,\mathrm{tar}}\left(\lambda \right)=\tau_{\mathrm{opt}}\frac{1}{4}M\left(\lambda \right){\left(\frac{d}{F/\#}\right)}^{2},$$

where M(λ) is the spectral radiant exitance with units $W\;m^{-2}\;\mu m^{-1}$; for a Lambertian source, M(λ)=πL(λ) with L(λ) in $W\;m^{-2}\;{\mathrm{sr}}^{-1}\;\mu m^{-1}$.

The single-channel SNR is

(14) $$\mathrm{SNR}=\frac{N_{\mathrm{sig}}}{\sqrt{N_{\mathrm{sig}}+N_{\mathrm{back}}+N_{\mathrm{dark}}+n_{\mathrm{read}}^{2}}},$$

The photoelectron counts $N_{\mathrm{sig}}$ can be expressed as:

$$N_{\mathrm{sig}}=T_{\mathrm{int}}\int_{\Delta \lambda }\frac{P_{\lambda ,\mathrm{tar}}\left(\lambda \right)\eta \left(\lambda \right)}{hc/\lambda }d\lambda ,\;N_{\mathrm{back}}=T_{\mathrm{int}}\int_{\Delta \lambda }\frac{P_{\lambda ,\mathrm{back}}\left(\lambda \right)\eta \left(\lambda \right)}{hc/\lambda }d\lambda ,$$

where $T_{\mathrm{int}}$ is the integration time, η(λ) is the detector quantum efficiency, and $P_{\lambda ,\mathrm{tar}}\left(\lambda \right)$ and $P_{\lambda ,\mathrm{back}}\left(\lambda \right)$ denote the spectral optical power incident on the detector for the target and background, respectively, after accounting for atmospheric transmission, optical throughput, and filter transmission. In Equation (14), $N_{\mathrm{dark}}$ represents the number of dark electrons accumulated during $T_{\mathrm{int}}$, and $n_{\mathrm{read}}$ is the rms readout noise in electrons per frame.

**Figure 3:**
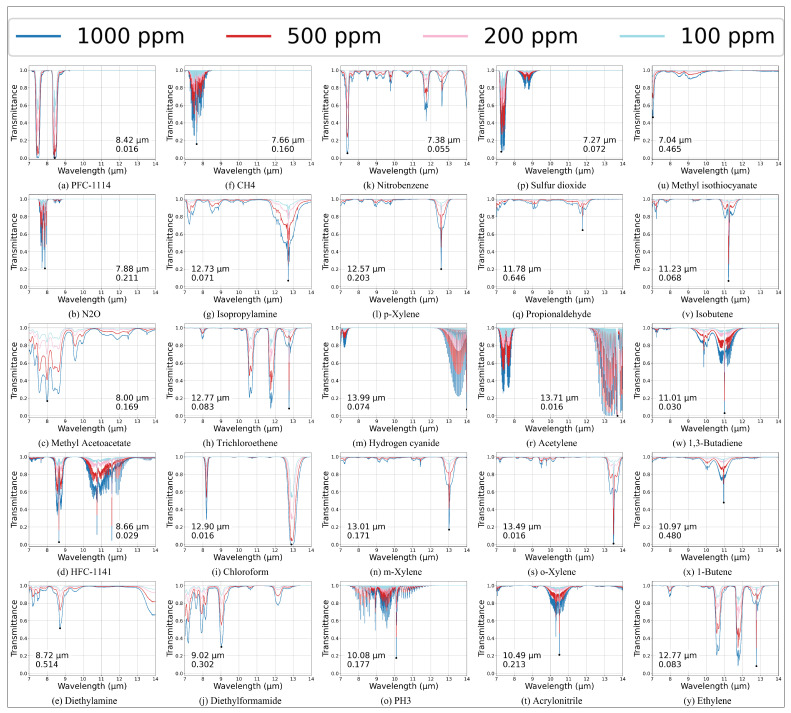
Transmittance spectra of 25 industrial gases within the wavelength range of 7–14 μm.

The wavelength dependence of gas transmittance, as described by Equation (12), gives rise to distinct spectral fingerprints for different gases. Figure 3 illustrates the computed transmittance spectra for 25 common industrial gases across the wavelength range of 7–14 μm. It facilitates future SNR assessments and path-integrated concentration retrieval, employing HITRAN data [35] and PNNL data [36] over a wide range of volume densities. The horizontal axis represents wavelength, and the vertical axis denotes transmittance. Red markers indicate the minimum transmittance $T_{\min}$ and the corresponding wavelength $\lambda_{\min}$ for each gas.

**Figure 4:**
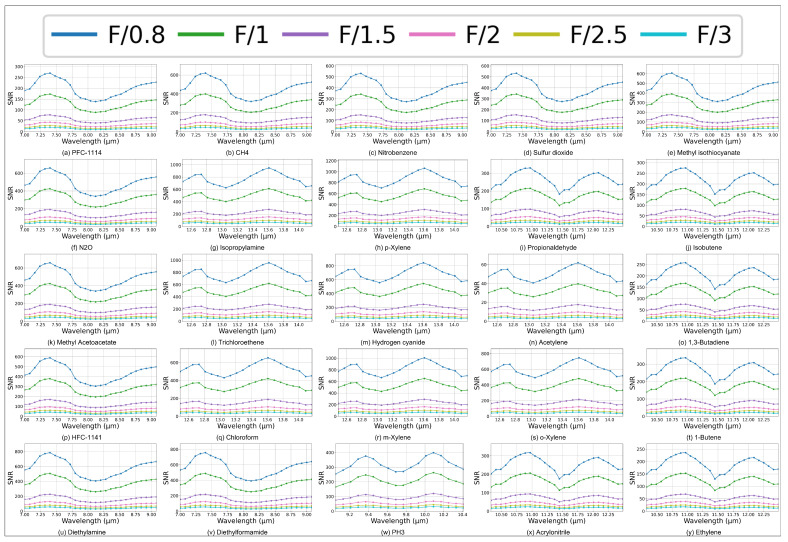
SNR as a function of wavelength across 7–14 μm for 25 gases at 100 ppm·m, contrasting F-numbers ranging from 0.8 to 3.

SNR values for different F-numbers at a path-integrated concentration of 100 ppm·m are compared to assess their effect on detection performance. Maintaining constant integration time, spectral bandwidth, and detector settings, a reduction in the F-number from 3.0 to 0.8 results in a monotonic increase in SNR across the range of 7–14 μm. Figure 4 illustrates that all gases display comparable spectral shapes with consistent peak locations, while variations in peak amplitudes arise from differences in transmittance spectra. At an F-number of 0.8, *p*-xylene exhibits the highest SNR, while gases that are comparatively challenging to detect, such as acetylene, PFC-114, ethylene, 1,3-butadiene, and isobutene, still attain SNRs on the order of $10^{2}$. For F-numbers ranging from 3.0 to 0.8, the detection of most gases yields SNR values between $10^{2}$ and $10^{3}$.

The smallest F-number considered yields the highest SNR across the spectral range. Nevertheless, reducing the F-number intensifies optical aberrations, increases the aperture size required, complicates system control, and tightens alignment tolerances. To achieve a balance between SNR and imaging quality, an F-number of 1 is selected as the design objective for the HRSMIS. The optical characteristics presented in Table 1 establish the design objectives and limitations for the comprehensive lens design and picture quality enhancement, which are discussed in the following sections. The two channels share the same fore telescope and observe the same scene, while their fields of view (FOV) differ due to the different effective focal lengths of the high-resolution and multispectral paths.

### 3.2. Optical Design of the HRSMIS

The HRSMIS is designed to capture multispectral images of various gas species within the 7–14 μm wavelength range, while maintaining detection capability for targets at distances of up to 1 km. The high-resolution channel employs an effective focal length of 110 mm, providing detailed imagery that preserves target edges and surface texture. The multispectral channel, with an effective focal length of 16.5 mm, divides the entrance aperture into 25 sub-apertures corresponding to distinct spectral channels for gas discrimination. According to the Johnson criterion, a 1.1 m-wide target located 1 km away covers approximately 8.1 pixels in the high-resolution channel and 1.2 pixels in the multispectral channel, demonstrating the system’s capability for remote gas detection and identification. The optical design process involves separate optimization of the two subsystems, followed by an integrated global optimization to ensure uniform and stable overall performance.

SNR modeling indicates that reducing the F-number significantly improves the detectability of gases with weak absorption features or low concentrations. An F-number of 1 is therefore adopted in the HRSMIS optical design, which inevitably introduces challenges such as increased optical aberrations, a larger entrance pupil diameter, and stricter manufacturing and alignment tolerances. To address the resulting complexity, the optimization strategy applies independent merit functions to the two subsystems. The telescope front section maintains uniform collimation across a wide FOV while achieving stringent distortion control. The high-resolution imaging subsystem is optimized using a weighted merit function that emphasizes aberration correction and maximizes optical throughput. The multispectral subchannels are designed to minimize interchannel crosstalk and sustain consistent imaging performance across all narrowband spectral channels.

**Figure 5:**
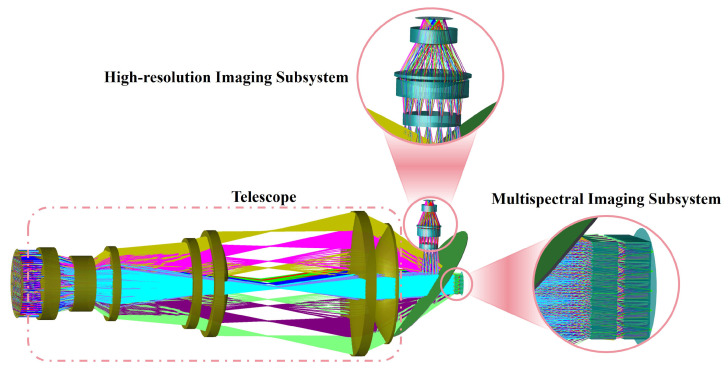
3D representation of the HRSMIS illustrating the telescope, high-resolution imaging subsystem, and multispectral imaging subsystem.

As shown in Figure 5, the HRSMIS optical configuration consists of a telescope, a high-resolution imaging subsystem, and a multispectral imaging subsystem. The three subsystems are designed and globally optimized under common image-plane and chief-ray-angle (CRA) constraints to ensure a unified boresight, a consistent FOV, and minimal distortion that allows effective error cancellation during image fusion. The following sections outline the design challenges and the corresponding optimization strategies employed for each subsystem.

#### 3.2.1. Telescope

**Figure 6:**
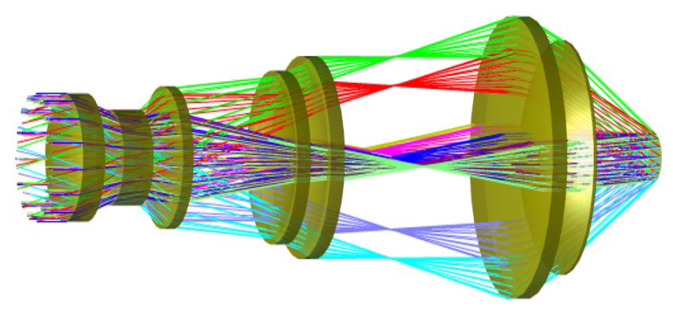
3D configuration of the telescope.

Figure 6 shows the optical structure of the telescope. The telescope generates a collimated beam with uniform irradiance across a wide FOV of 20°×20° and aligns the exit pupil to feed both the high-resolution and multispectral imaging subsystems. A beam splitter is positioned at the telescope’s exit pupil to divide the collimated beam between the two imaging paths.

To mitigate axial and lateral chromatic aberration, the telescope’s image-quality optimization function assigns greater weighting to the band-edge wavelengths at 7 μm and 14 μm, as well as to several representative in-band points. Beam-splitter placement and mechanical clearance for the downstream subsystem are achieved by treating the back focal length (BFL) as a design variable, which converged to an optimized value of 196.23 mm after multiple iterations. The beam splitter is positioned at the telescope’s exit pupil to reduce vignetting and minimize CRA inconsistencies between the two imaging subsystems. The principal optical parameters of the telescope are summarized in Table 2.

#### 3.2.2. High-Resolution Imaging Subsystem

**Figure 7:**
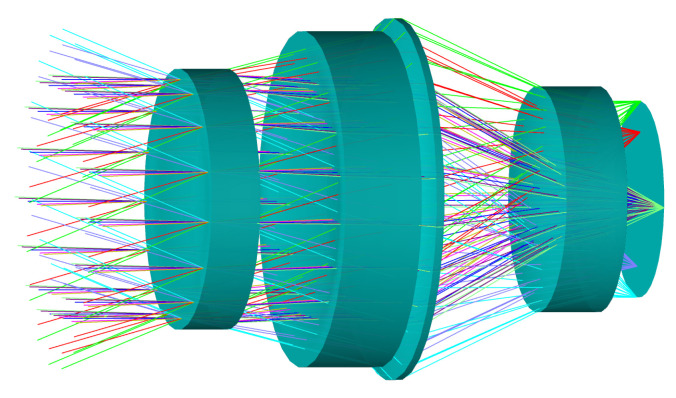
3D configuration of the high-resolution imaging subsystem.

The optical layout of the high-resolution imaging subsystem is shown in Figure 7. The subsystem comprises four lenses that provide high transmittance and effective aberration control, with a FOV of 39.6°×22.9°. To mitigate high-order aberrations induced by the large relative aperture, the lenses incorporate even-order aspheric surfaces, and their curvatures and spacings are optimized as variable parameters during the design process. Chromatic aberration is further reduced through the coordinated selection of materials, combining HWS7, HWS5, KBr, and GaAs to exploit complementary dispersion characteristics across the 7–14 μm spectral range. The optical specifications of the subsystem are summarized in Table 3. The entrance pupil is designed to be identical in size and precisely aligned with the telescope’s exit pupil, ensuring accurate pupil matching and geometric consistency between the high-resolution and multispectral imaging subsystems.

#### 3.2.3. Multispectral Imaging Subsystem

**Figure 8:**
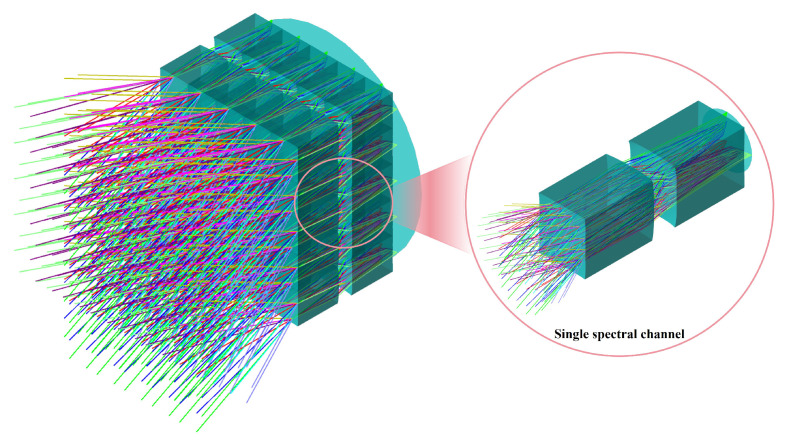
3D representation of the multispectral imaging subsystem, accompanied by a localized magnified illustration of an individual spectral channel.

The multispectral imaging subsystem receives the collimated beam from the front telescope and forms the corresponding image. As shown in Figure 8, two MLAs arranged in a 5×5 configuration divide the telescope’s exit pupil into 25 sub-imaging apertures. The paired MLAs work cooperatively to correct optical aberrations and achieve high image quality across the entire FOV. Each subchannel is independently imaged and equipped with a dedicated narrowband filter. The spectral bandwidth of each filter is approximately 80 nm, with a cut-off optical density of OD4. The center wavelength of each filter is aligned with the absorption peak of its target gas, enabling the acquisition of 25 spectral images of the same scene. To ensure compatibility with the front telescope, the CRA in object space for each sub-spectral channel is constrained during MLA optimization to match that of the telescope, thereby maintaining uniform imaging across all channels. To minimize central-wavelength shifts caused by variations in the incidence angle on the filters, the optimization process also strictly limits the CRA in the image space. The optimized optical parameters for a single imaging unit within the MLA of the multispectral imaging subsystem are summarized in Table 4.

## 4. Imaging Performance

To comprehensively evaluate the imaging performance of HRSMIS, a range of standard optical performance metrics is employed, including the spot diagram, wavefront aberration, distortion, PSF, and modulation transfer function (MTF). The spot diagram assesses imaging performance within the framework of geometric optics by plotting ray intercepts on the image plane corresponding to different pupil coordinates. This visualization illustrates the energy distribution caused by aberrations while neglecting diffraction effects. To complement this geometric-optics-based evaluation, wavefront aberration is introduced from the viewpoint of physical optics to provide a more complete assessment of image quality. It quantifies the optical path difference (OPD) between the emergent wavefront and an ideal spherical reference, typically represented using Zernike polynomial decomposition. Imaging quality is generally considered satisfactory when the peak-to-valley (PV) OPD is less than λ/4. Distortion describes the mapping error of image height arising from FOV-dependent variations in magnification, and the system requires that relative distortion remain below 3% across the full FOV. The PSF, obtained through diffraction integration, characterizes the irradiance distribution in the image plane and serves as a verification reference for MTF and wavefront analyses. The MTF evaluates the system’s ability to transfer spatial information and is mathematically equivalent to the Fourier transform of the PSF, as defined in Section 2. For HRSMIS, the MTF is assessed at a spatial frequency of 33 cycles/mm, corresponding to the Nyquist frequency defined in Equation (15). Together, these metrics provide a multidimensional assessment of image quality, ensuring that HRSMIS achieves the desired imaging performance.

(15) $$N=\frac{1000}{2d}\;\left(\mathrm{cycles}/\mathrm{mm}\right),$$

where *d* is the pixel pitch in micrometres.

### 4.1. Subsystem Performance

#### 4.1.1. Telescope Performance

**Figure 9:**
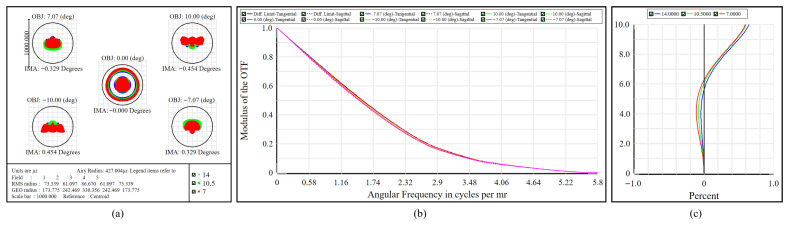
Performance of the telescope subsystem. (**a**) Spot diagram. (**b**) MTF. (**c**) Distortion curves.

The performance of the telescope subsystem is presented in Figure 9. Figure 9a illustrates that the RMS spot radius throughout the entire FOV ranges from 61–87 μrad, which is within the Airy disk radius of 427 μrad. The symmetry of the spot patterns suggests effective management of astigmatism and coma. In reference to the MTF illustrated in Figure 9b, the tangential and sagittal curves closely align with the diffraction limit from 0 to 5.8 cycles/mrad, reaching unity at low spatial frequencies and subsequently exhibiting a smooth decline at higher frequencies, indicating overall performance near the diffraction limit. Figure 9c indicates that the relative distortion at the maximum FOV is 0.691%, which is well within the 3% specification for the telescope. The results demonstrate that the telescope delivers near-diffraction-limited image quality with minimal distortion across the full spectral range and FOV, providing a high-quality collimated beam to the subsequent imaging subsystems.

#### 4.1.2. High-Resolution Imaging Subsystem Performance

**Figure 10:**
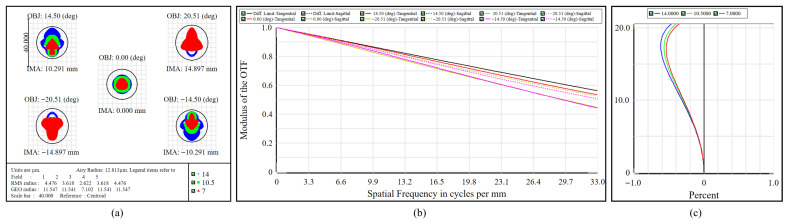
Performance of the high-resolution subsystem. (**a**) Spot diagram. (**b**) MTF. (**c**) Distortion curves.

The high-resolution imaging subsystem is designed to achieve superior spatial resolution, necessitating precise optimization of image quality. Figure 10a shows the polychromatic spot diagram, where the RMS spot radii range from 2.622 μm to 4.476 μm, which are significantly lower than the Airy disk radius of 12.813 μm. Similarly, the geometric radii of 7.102–11.547 μm also fall below the Airy limit, suggesting effective aberration suppression. As illustrated in Figure 10b, the MTF remains above 0.44 at 33 cycles/mm across the entire FOV, and the on-axis response nearly coincides with the diffraction-limited reference, confirming high image contrast and excellent optical performance. Figure 10c shows that the maximum relative distortion value is 0.572% at a FOV angle of 20.51°, satisfying the criterion of keeping distortion below 3%. The high-resolution imaging path maintains stable image quality and high contrast throughout the working band and the entire FOV.

#### 4.1.3. Multispectral Imaging Subsystem Performance

**Figure 11:**
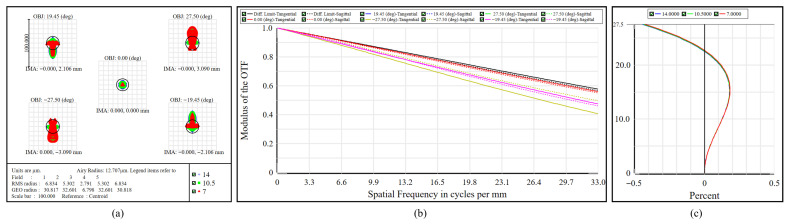
Performance of the multispectral subsystem. (**a**) Spot diagram. (**b**) MTF. (**c**) Distortion curves.

The multispectral imaging channel is required to display 25 images simultaneously, necessitating precise control of spectral differentiation. Figure 11a shows the polychromatic spot diagram, where the RMS spot radii range from 2.791 μm to 6.834 μm, all well below the corresponding Airy disk radius at each wavelength. The compact, near-circular spot patterns indicate accurate focusing and effective aberration correction. The MTF curves are illustrated in Figure 11b. At 33 cycles/mm, the MTF surpasses 0.40 in all FOVs, with the on-axis trace closely aligning with the diffraction-limited reference, indicating a high level of overall contrast. Regarding distortion, Figure 11c shows a maximum relative value of 0.620% at a FOV of ±27.5°, satisfying the criterion that distortion remains below 3%.

### 4.2. Integrated System Performance

#### 4.2.1. High-Resolution Channel

**Figure 12:**
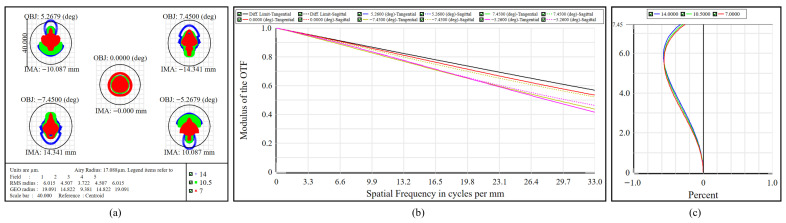
Integrated system performance of the high-resolution channel. (**a**) Spot diagram. (**b**) MTF. (**c**) Distortion curves.

The imaging performance of the high-resolution channel is presented in Figure 12. Figure 12a depicts the polychromatic spot diagram over 7–14 μm. Across the full FOVs, the RMS spot radii fall within 3.722–6.015 μm and remain well below the corresponding 17.088 μm Airy disk radius, yielding compact, energy-concentrated spots consistent with effective suppression of aberrations. Figure 12b shows that at 33 cycles/mm, the MTF exceeds 0.40 for all FOVs, thereby sustaining stable image contrast. As for geometric fidelity, Figure 12c indicates a maximum relative distortion of 0.574% near a FOV angle of ±7.45°, with the distortion remaining predominantly negative across the FOV. At the system level, a distortion-balancing scheme is applied to control residual geometric discrepancies, thereby facilitating accurate cross-channel registration and subsequent data fusion. The telescope exhibits positive distortion, whereas the high-resolution and multispectral subsystems introduce negative distortion, resulting in partial mutual compensation of their effects. After joint optimization, the high-resolution subsystem maintains image quality near the diffraction limit and preserves a low level of distortion.

#### 4.2.2. Multispectral Channel

Although the telescope is optimized to deliver good imaging performance over the full operating band, satisfactory broadband performance does not necessarily guarantee uniformly high image quality at every individual narrowband channel; therefore, we subsequently report the imaging metrics for all 25 narrowband channels to rigorously assess the HRSMIS.

**Figure 13:**
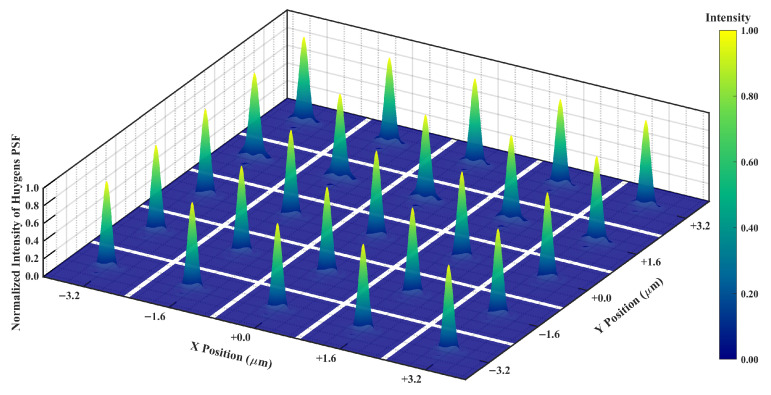
Huygens PSF of the 25 multispectral channels.

Figure 13 demonstrates the Huygens PSF for the multispectral channel within the integrated system. The PSFs display a nearly circular symmetric main lobe with concentrated energy and controlled dispersion, weak sidelobes, and the absence of spurious artifacts. The main lobe width and peak height exhibit consistency across channels, suggesting stable imaging kernel behavior throughout the spectral bands. The Strehl ratio for each channel approaches unity, consistent with the prior MTF and wavefront analyses, thus validating effective aberration suppression and stable focusing and frequency response.

**Figure 14:**
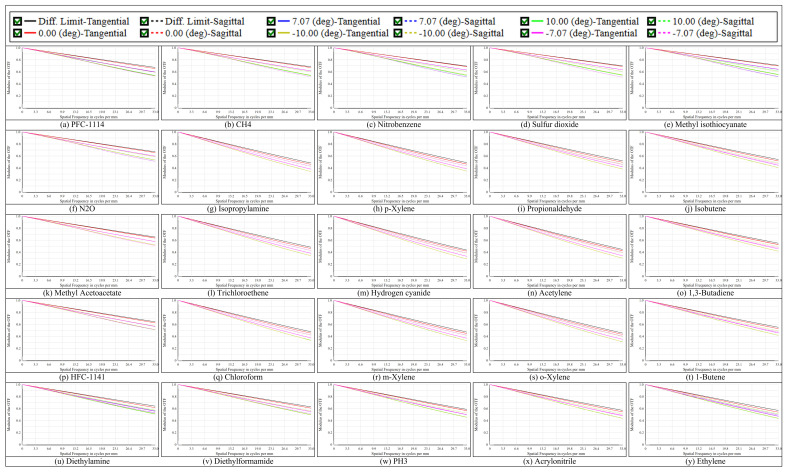
The MTF for different channels at the FOVs between −10° and 10°.

Figure 14 displays the MTF curves for the multispectral channel within the integrated system, utilized for assessing spatial frequency transfer characteristics. All channels exhibit elevated modulation at low to mid spatial frequencies, with each curve typically aligning with the diffraction limit reference. At 33 cycles/mm, the MTF values across the FOV generally range from 0.29 to 0.70. The 7.00–7.08 μm channels exhibit optimal performance, with MTFs surpassing 0.50 throughout the entire FOV, while the longwave end channels at 13.95–14.03 μm maintain values above 0.29 across the FOV. All MTF curves exhibit continuity and smoothness without anomalous oscillations, demonstrating consistency across channels and effective management of optical prescription and alignment errors. The balanced frequency response ensures reliable preservation of spatial resolution and structural detail in multispectral channel image fusion.

**Figure 15:**
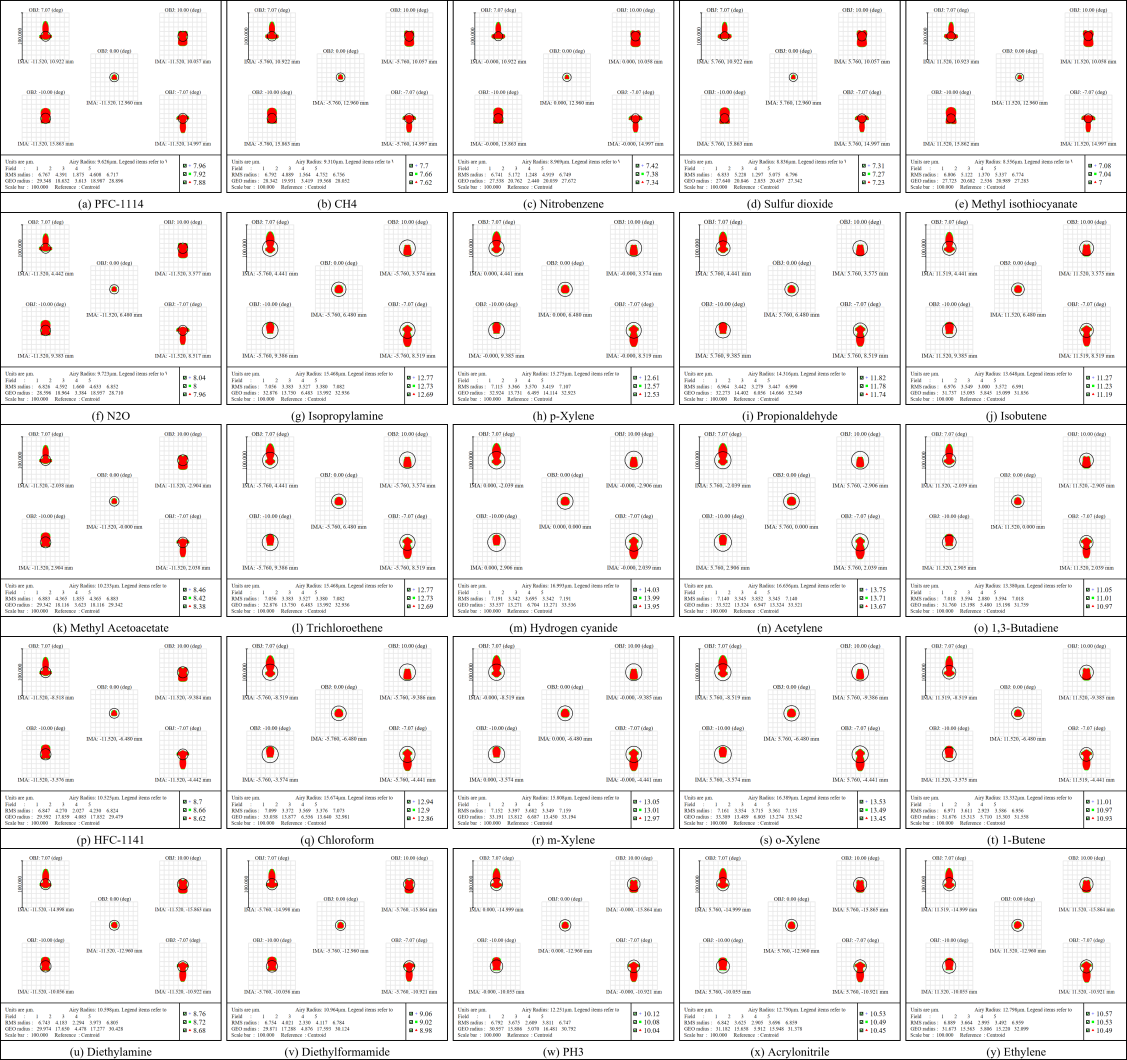
The spot diagram for different channels at the FOVs between −10° and 10°.

The spot diagram is examined to evaluate the performance of the HRSMIS. Figure 15 shows the spot diagrams for the multispectral channel within the integrated system, utilized for evaluating focusing performance. Each subchannel is ray traced at its central wavelength; distinct colors represent varying wavelengths, and the red circle indicates the Airy disk radius for that band. All 25 subchannels exhibit RMS spot radii that fall within their corresponding Airy disks. The spots exhibit compact, nearly circular shapes, lacking comet tails, tangential and sagittal separation, and abrupt changes in aberration, which suggests strong interchannel consistency. For the 13.95–14.03 μm subchannels within ±10° FOV, the RMS spot radius varies from 3.695 to 7.191 μm, whereas the Airy disk radius is 16.993 μm. In the 7.00–7.08 μm subchannels, the RMS spot radius varies between 1.370 and 6.806 μm, while the Airy disk radius is 8.556 μm. All subchannels demonstrate stable focusing performance and spatial uniformity across all narrowband spectral channels and the FOV, thus establishing a dependable optical foundation for subsequent image fusion and gas identification.

**Figure 16:**
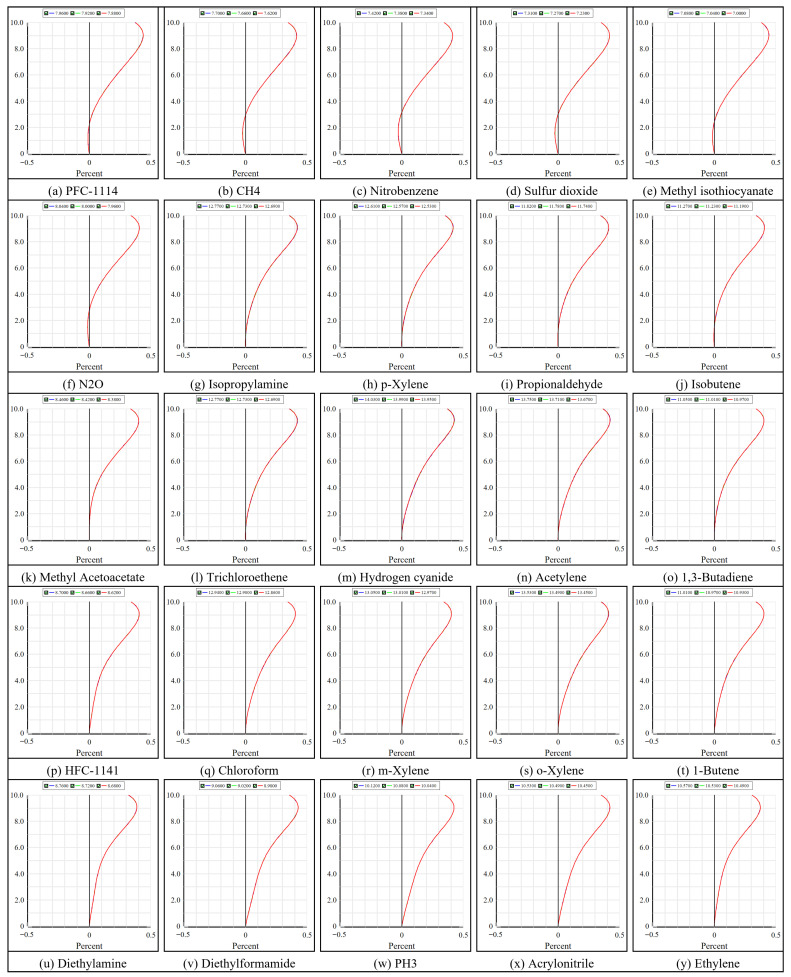
The distortion curves for different channels at the FOVs between 0° and 10°, in which the horizontal axis expresses the distortion in %, and the vertical axis gives the half FOV.

Geometric mapping performance is evaluated through the distortion distribution curves of the multispectral channel, as shown in Figure 16. All channels have negative distortion with a continuous, symmetric variation throughout the FOV, and no abrupt variations are seen. At the boundary of the FOV, the deviation for each channel is confined to ±0.6%. In the maximum FOV, the 7.00–7.08 μm channel exhibits the highest absolute magnitude at 0.4587%, while the 10.49–10.57 μm channel displays the lowest at 0.3752%. Interchannel disparities are negligible, signifying consistent geometric behavior among channels. The HRSMIS therefore, meets the design objective of maintaining distortion below 3%, providing a reliable geometric basis for subsequent multichannel registration and fusion reconstruction.

**Figure 17:**
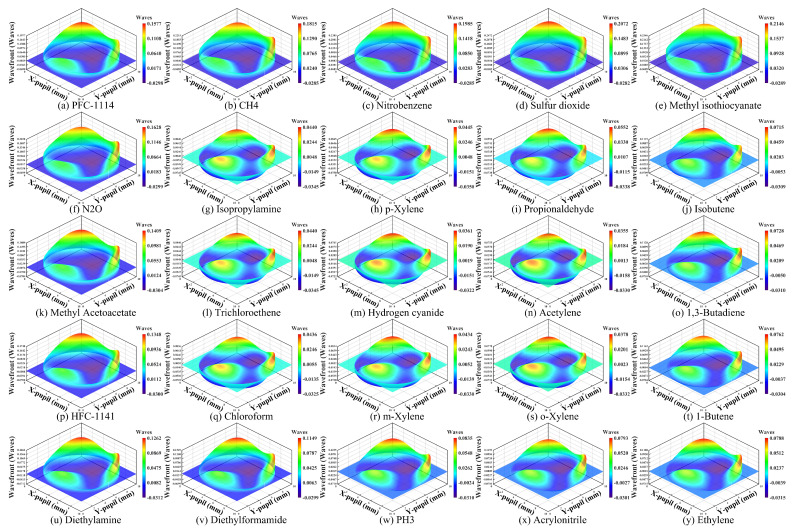
The wavefront maps for different channels at 10°, in which the x and y axes represent the coordinate positions of the pupil.

Wavefront aberrations are analyzed to confirm that HRSMIS meets the requirements for high imaging accuracy and low aberrations. Figure 17 illustrates the wavefront maps corresponding to the multispectral channel within the integrated system. The wavefronts exhibit a generally smooth and approximately symmetric profile, with the primary variation focused near the pupil center, while aberrations in the edge region are effectively minimized. Several subchannels display minor third-order coma or astigmatism at the aperture periphery; however, no abrupt discontinuities are detected. The peak PV value is illustrated in Figure 17e for the 7.00–7.08 μm subchannels, recorded at 0.2435 λ, which is consistently below 0.25 λ. The results demonstrate a high level of uniformity across channels in terms of wavefront quality and aberration control, establishing a reliable optical foundation for future multichannel fusion, geometric registration, and imaging consistency analysis.

**Figure 18:**
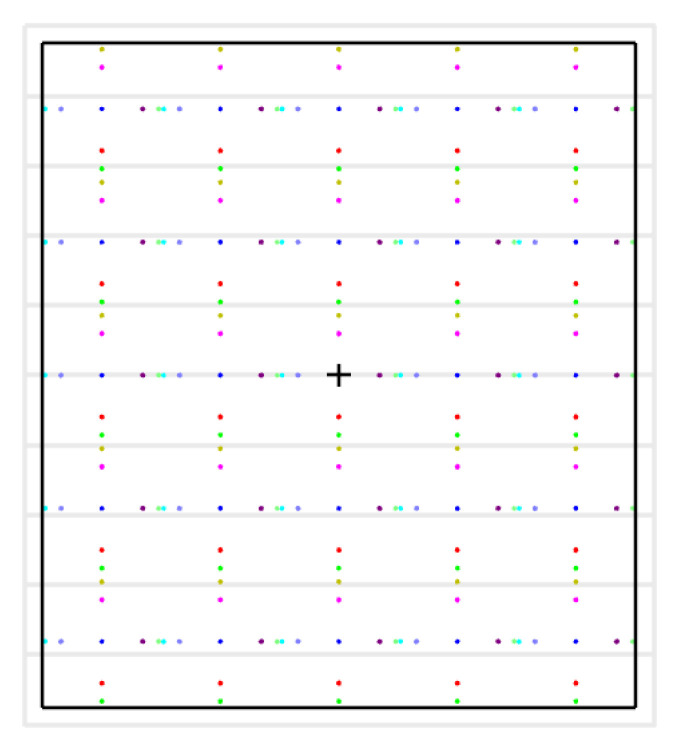
Footprint diagram of the HRSMIS.

The system’s pivotal capability is the concurrent acquisition of 25 pictures on a single FPA. Figure 18 illustrates the footprint configuration of HRSMIS for all 25 channels. A 25-pixel spacing is maintained between multispectral images, ensuring complete subimage integrity and eliminating interchannel crosstalk.

## 5. Tolerance Analysis

To ensure the manufacturability, assembly accuracy, and testing performance of HRSMIS, tolerance analysis is conducted based on sensitivity analysis, followed by a Monte Carlo simulation. A Gaussian error model is employed to introduce random perturbations to parameters such as the radius of curvature, element thickness, surface decentering and tilt, as well as the refractive index and Abbe number. The resulting effects on imaging performance metrics, including the MTF, are statistically evaluated. The tolerance ranges are iteratively refined through repeated analyses, where strict limits are assigned to high-sensitivity parameters, while low-sensitivity parameters are allowed greater flexibility. The final optimized tolerance budget is presented in Table 5. Considering the distinct optical configurations of the multispectral and high-resolution channels, the tolerance data are specified separately for each subsystem.

**Figure 19:**
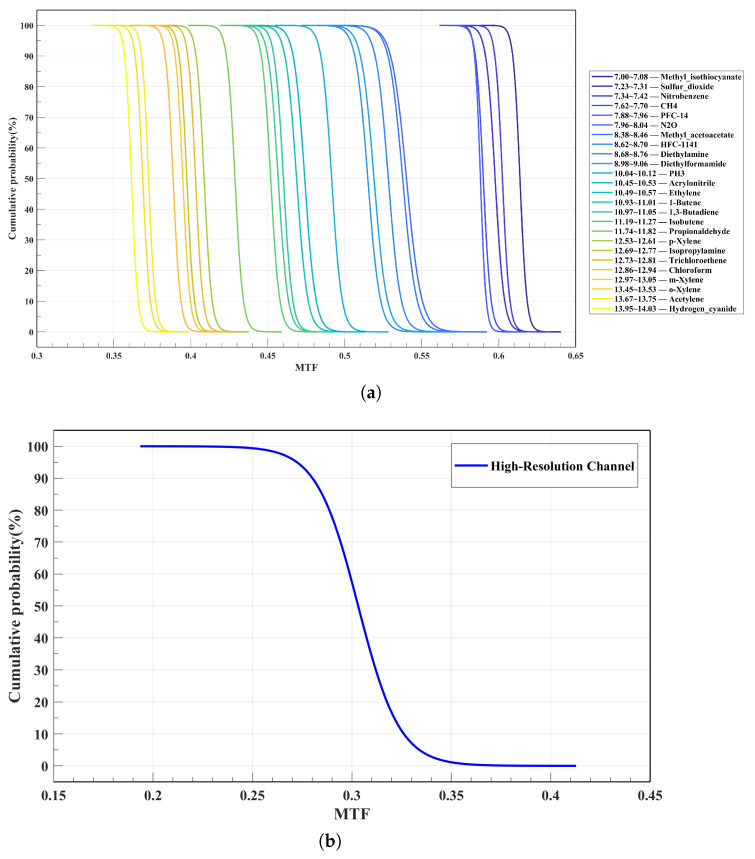
Cumulative probability curves of MTF at 33 lp/mm. (**a**) Multispectral channels. (**b**) High-resolution channel.

Taking into account manufacturing and alignment tolerances, the cumulative MTF probability curves in Figure 19a indicate that the MTF reduction remains under 0.10 for all narrowband spectral channels, with 80% of channels exhibiting a reduction below 0.06. The channels most responsive to tolerances are 7.23–7.31 μm and 7.34–7.42 μm, exhibiting maximum MTF reductions of 0.0571 and 0.0570, respectively. Upon applying tolerances, the resultant MTF values converge to 0.5691 and 0.5799. The cumulative probability curves are steep and concentrated toward the upper end, indicating that process variations primarily cause minor fluctuations in MTF rather than a broad degradation of imaging performance. At the 90% cumulative probability level, the MTF reaches 0.595 for the 7.00–7.08 μm channel and 0.344 for the 13.95–14.03 μm channel. For the 7.00–7.08 μm channel, the nominal MTF value is 0.629, with an expected reduction of 0.035. For the 13.95–14.03 μm channel, the nominal value is 0.380, with an acceptable decrease of 0.031, resulting in 0.349. The MTF remains above 0.59 for the 7.00–7.08 μm channel and above 0.34 for the 13.95–14.03 μm channel. Figure 19b illustrates the cumulative probability curves for the MTF of the high-resolution imaging channel at the Nyquist frequency of 33 lp/mm, derived from Monte Carlo analysis. At the 90% cumulative probability level, the average MTF across the whole FOV is 0.204. Overall, the analysis confirms that the HRSMIS preserves satisfactory optical performance and imaging quality within the defined tolerance limits.

## 6. Discussion

A Longwave Infrared High-Resolution Snapshot Multispectral Imaging System (HRSMIS) is proposed to integrate spatial detail and spectral discrimination for real-time monitoring of gaseous emissions. The HRSMIS captures high-resolution images across 25 narrow spectral bands within the 7–14 μm region in a single exposure. A shared fore-optical path followed by a beam splitter directs the incident radiation into two synchronized imaging channels. The multispectral channel adopts an aperture-division configuration, combining dual 5×5 microlens arrays with a corresponding narrowband filter array to produce 25 discrete spectral images, whereas the high-resolution channel records fine spatial information for super-resolution image fusion. The optical configuration, characterized by an F-number of 1, achieves an optimized balance among optical throughput, detection sensitivity, and spatial resolution, enabling kilometer-scale gas detection and supporting rapid, high-sensitivity identification of hazardous gases in open environments, together with video-rate visualization of diffusion dynamics and quantitative retrieval of concentration distributions.

The optical performance indicates that HRSMIS delivers sufficient image quality for gas species differentiation across the 7–14 μm spectral range and throughout the entire FOV. For the high-resolution channel, the polychromatic RMS spot radius varies from 2.622 μm to 4.476 μm, compared with an Airy disk radius of 12.813 μm. The MTF at 33 cycles/mm remains above 0.44 across the FOV, with a maximum distortion of 0.572%. In the multispectral channel, all 25 subchannels exhibit RMS spot radii smaller than their corresponding Airy disk radii. The MTF at 33 cycles/mm generally ranges from 0.29 to 0.70. The wavefront PV is consistently below 0.25 λ, and the maximum distortion at the maximum FOV is 0.620%. Huygens PSF analysis reveals compact, nearly circular main lobes with weak sidelobes and uniform peak intensities across all subchannels. Adjacent subimages are separated by 25 pixels, showing no interchannel crosstalk or missing subimages.

Tolerance analysis is performed using 1000 sensitivity analyses and 1000 Monte Carlo simulations. The cumulative probability curves of the MTF for the multispectral channel at 33 lp/mm indicate that the reduction remains below 0.10 for all channels and below 0.06 for approximately 80% of them. At the 90% cumulative probability level, the MTF reaches 0.595 for the 7.00–7.08 μm range and 0.344 for the 13.95–14.03 μm range, confirming that the MTF stays above 0.59 and 0.34 for the corresponding subchannels. For the high-resolution channel, Monte Carlo cumulative probability curves at 33 lp/mm show an average MTF of 0.204 across the full FOV at the 90% confidence level. The results verify that the optical design provides adequate stability against manufacturing and alignment tolerances, ensuring that the system maintains the expected imaging performance under practical fabrication and assembly conditions.

## 7. Conclusions

Timely localization of gas emissions, identification of species, and characterization of dispersion patterns are critical for effective monitoring in complex and dynamically changing environments. Conventional dispersion spectrometers and filter wheel multispectral cameras that sequentially record spectral and spatial information exhibit limited temporal resolution and cannot capture transient plume dynamics. A Longwave Infrared High-Resolution Snapshot Multispectral Imaging System (HRSMIS) is proposed, capable of simultaneously acquiring 25 high-resolution multispectral images of the same target within a millisecond-level single exposure. The system enables video-rate gas imaging with strong real-time performance and can identify a wide range of gaseous species. HRSMIS provides detailed spatial distribution data and supports quantitative retrieval of gas concentration. The optical performance and manufacturability have been verified through comprehensive design analysis and statistically based tolerance evaluation. The system maintains compact spot regions within the Airy disk, preserves the MTF at 33 cycles/mm within the specified range for both channels, minimizes distortion across the full FOV, and keeps the multispectral wavefront PV below 0.25 λ. Monte Carlo simulations further confirm that the system maintains the expected imaging performance under realistic fabrication and assembly conditions. The results demonstrate the potential of HRSMIS to enhance real-time infrared monitoring of atmospheric pollutants and contribute to advanced environmental observation and emission control applications.

Future work will concentrate on constructing prototype instruments and establishing rigorous spectral and radiometric calibration frameworks, alongside the development of neural-network-guided super-resolution reconstruction algorithms and comprehensive gas detection and identification experiments under representative environmental conditions.

## Figures and Tables

**Table 1:** HRSMIS optical design parameters.

System	Focal Length (mm)	F-Number	Magnification	Field of View (FOV)
Telescope	–	1	2.75×	20°×20°
High-resolution subsystem	40	1	–	39.6°×22.9°
Multispectral subsystem	6	1	–	55°×55°
High-resolution channel	110	1	–	14.82°×8.42°
Multispectral channel	16.5	1	–	20°×20°

**Table 2:** Telescope lens data: surface curvatures, thickness, and materials.

Lens	Front Surface Curvature (mm)	Back Surface Curvature (mm)	Thickness (mm)	Material
1st	151.7736	210.7378	42.4606	KR5S
2nd	916.1809	132.5733	44.1268	CdSe
3rd	3546.4314	−291.9926	24.8760	IRG25
4th	−146.8931	−250.9555	15.2536	KBr
5th	−173.1107	−148.7792	31.7519	AgCl
6th	513.7720	−3162.6789	40.5067	IRG23
7th	130.7685	129.5465	10.6859	Ge

**Table 3:** High-resolution imaging subsystem lens data: surface curvatures, thickness, and materials.

Lens	Front Surface Curvature (mm)	Back Surface Curvature (mm)	Thickness (mm)	Material
1st	−113.1185	−165.5318	9.1516	HWS7
2nd	223.0038	−48.7077	4.0000	KBr
3rd	−63.2260	177.4220	11.0001	HWS5
4th	−53.7236	−39.6538	9.8138	GaAs

**Table 4:** Multispectral imaging subsystem lens data: surface curvatures, thickness, and materials.

Lens	Front Surface Curvature (mm)	Back Surface Curvature (mm)	Thickness (mm)	Material
1st	35.8733	−126.1961	8.7528	Ge
2nd	19.3217	21.2755	8.4022	Ge

**Table 5:** Specifications of the tolerance allocation scheme.

Regime	Radius of Curvature Tolerance (Aperture Units)	Thickness Tolerance (mm)	Eccentric Distance of Lens Surface (mm)	Inclination of Lens Surface (°)	Inclination of Lens Element (°)	Refractive Index Tolerance	Abbe Number Tolerance/%
A	1	±0.01	±0.005	±0.001	±0.001	±0.0005	±0.5
B	2	±0.0375	±0.01	±0.0167	±0.0167	±0.001	±0.1
C	3	±0.03	±0.03	±0.017	±0.017	±0.001	±1

Regime–band mapping (unit: μm). A: 7.00–7.08, 7.23–7.31, 7.34–7.42, 7.62–7.70, 7.88–7.96; B: 7.96–8.04, 8.38–8.46, 8.62–8.70, 8.68–8.76, 8.89–9.06, 10.04–10.12, 10.45–10.53, 10.49–10.57, 10.93–11.01, 10.97–11.05, 11.19–11.27, 11.74–11.82, 12.53–12.61, 12.69–12.77, 12.73–12.81, 12.86–12.94, 12.97–13.05, 13.45–13.53, 13.67–13.75, 13.95–14.03; C: high-resolution imaging channel.

## Data Availability

The data are not publicly available due to confidentiality agreements.

## Abbreviations

The following abbreviations are used in this manuscript:

LWIR	Long-wave Infrared
FTIR	Fourier transform infrared spectroscopy
SNR	Signal-to-Noise Ratio
HRSMIS	High-Resolution Snapshot Multispectral Imaging System
NETD	Noise Equivalent Temperature Difference
MLA	Microlens Array
FPA	Focal Plane Array
PSF	point spread Function
LSI	Linear Shift-Invariant
IFOV	Instantaneous Field of View
GSD	Ground Sampling Distance
FOV	Field of View
MTF	Modulation Transfer Function
BFL	Back Focal Length
CRA	Chief Ray Angle
OPD	Optical Path Difference
PV	Peak-to-Valley
RSS	Root-Sum-Square
